# Association of Cardiorespiratory Fitness and Cognitive Function with Psychological Well-Being in School-Aged Children

**DOI:** 10.3390/ijerph19031434

**Published:** 2022-01-27

**Authors:** Weiyun Chen, Xiangli Gu, Jun Chen, Xiaozan Wang

**Affiliations:** 1 School of Kinesiology, University of Michigan, Ann Arbor, MI 48109, USA; 2 Department of Kinesiology, University of Texas at Arlington, Arlington, TX 76109, USA; xiangli.gu@uta.edu; 3 School of Physical Education and Health, East China Normal University, Shanghai 200241, China; michaelsara2011@163.com; 4 Key Laboratory of Adolescent Health Assessment and Exercise Intervention of Ministry of Education, East China Normal University, Shanghai 200241, China

**Keywords:** well-being, aerobic fitness, cognition, concentration performance, attention span, attention accuracy

## Abstract

Background: Promotion of psychological well-being (PWB) is an emerging social, educational, and health objective, especially for school-aged children. Few studies have examined key correlates and determinants of PWB in school-aged children. This study aimed to examine associations of cardiorespiratory fitness and cognitive function with psychological well-being in school-aged children. Methods: The study participants were 752 fourth-grade students (mean _age_ = 9.61 years, SD = 0.608) recruited from six elementary schools. Students took the Progressive Aerobic Cardiovascular Endurance Run^®^ test to assess their cardiorespiratory fitness, and the d2 Test of Attention to assess concentration performance, attention span, and attention accuracy. They also completed the Warwick–Edinburgh Mental Well-Being Scale to assess their psychological well-being (PWB). After removing missing values and outliers from the original data set, the final data set, consisting of 689 cases (370 boys vs. 319 girls), was used for data analysis. Data were analyzed by means of descriptive statistics, bivariate correlation, multiple linear regression models, and independent sample *t*-tests. Results: The results indicated that cardiorespiratory fitness and cognitive function are significant correlates of PWB (*r* = −0.069, *r* = 0.161). Further, the results found that cardiorespiratory fitness, concentration performance, attention span, and attention accuracy were significantly collective predictors of psychological well-being (*F* = 13.299, *p* = 0.000), accounting for 12% of the total variance. Cardiorespiratory fitness was the most significantly individual predictor of PWB (*β* = 0.174, *p* = 0.000), followed by the attention accuracy (*β* = −0.090, *p* = 0.031). The Welch’s tests revealed that the high-PWB group scored significantly higher than the low-PWB group in cardiorespiratory fitness, concentration performance, and attention accuracy (*t* = 4.093, *p* = 0.000, Cohen’s *d* = 0.310; *t* = 3.340, *p* = 0.001, Cohen’s *d* = 0.256; *t* = −2.958, *p* = 0.003, Cohen’s *d* = 0.130). Conclusions: Cardiorespiratory fitness and cognitive function are significant correlates and predictors of PWB among school-aged children. The students with a higher level of psychological well-being showed a higher cardiorespiratory fitness, concentration performance, and attention accuracy compared to the lower level of PWB group.

## 1. Background

Promotion of psychological well-being (PWB) is an emerging social, educational, and health objective. More recently, promoting PWB in childhood has received increasing attention in public health and school education. PWB refers to a positive mental, social, and emotional state that allows individuals to pursue happiness, life satisfaction, personal flourishing, and a meaningful life [1,2,3]. Based on two predominant perspectives of PWB, the hedonic approach focuses on affective-emotional aspects of functioning such as subjective happiness, positive and negative affect, and life satisfaction [2,4]. The eudaimonic facet highlights cognitive–evaluative and psychosocial aspects of functioning, including self-acceptance, positive relations with others, autonomy, environmental mastery, purpose in life, and personal growth [1]. PWB connotes an individual’s optimal psychosocial functioning [5].

A growing body of studies have shown that different facets of PWB are associated with positive health outcomes [6,7]. For example, a longitudinal study found that low levels of negative affect were independently associated with long-term survival among 4888 men and women aged 18–81 years [7]. Another longitudinal study reported that a higher level of life purpose was related to a lower level of death over a two-year period after controlling for psychological distress among of 7675 older women [8]. PWB are found to be consistent protective factors of cardiovascular diseases [6,9]. In addition, studies indicate that enhanced PWB play essential roles in developing positive personal attributes, building social and emotional skills and competency, establishing positive social relationships, and enhancing academic behaviors and achievement in adolescents [10,11]. For instance, a cross-sectional study found that frequent positive emotions were linked to greater academic engagement, while negative emotions were associated with less student engagement among 293 students in grades 7–10 [10]. In a longitudinal study of 300 middle school students in grades 6–9, Suldo et al. [11] reported that the students with both average and high subjective well-being and low psychopathology showed best academic behaviors (i.e., attendance) and academic performance (i.e., grade point average and math skills) over time. PWB is conducive to promoting physical and mental health among adolescent and adult populations [1,2,12]. Given the short- and long-term impacts of PWB on an individual’s physical and mental health and academic success, understanding potential correlates/determinants of PWB in childhood is an urgent public health priority [2,3,13].

To date, studies on correlates of PWB, such as cognitive function and cardiorespiratory fitness, in school-aged children have remained a largely untapped territory. Cognitive function refers to multiple mental processes such as attention, concentration performance, memory, problem solving, and decision making [14]. Attention and concentration performance are core components of cognitive function [15]. Attention and concentration performance are key contributors to successful academic performance, adaptive behaviors, and daily life functioning in youth [16]. In a conceptual model, Lubans et al. [14] proposes that cognitive function provides core foundation for developing and establishing PWB. However, research on relationship of attention and concentration performance with PWB in youth is still scarce. Only one early study examined the associations between cognitive function, depression, and PWB among 11,234 older adults with the mean age of 65.2 years [17]. The study found that greater PWB was significantly associated with better cognitive function after controlling for depressive symptoms [17].

Cardiorespiratory fitness in childhood is a key marker of health [7,18]. It refers to an individual’s capacity of heart, blood vessels, and lungs to transport oxygen to skeletal muscles for their efficient use of oxygen in order to perform prolonged exercises without overly fatiguing [7,18]. Children with healthy cardiorespiratory fitness tend to be healthier adults and to have greater longevity [7,18]. Healthy levels of cardiorespiratory fitness are associated with lower risk for developing premature cardiovascular disease, type 2 diabetes, and high blood pressure at younger ages [7,18]. Healthy cardiorespiratory fitness is linked to better academic achievement and decreased mental stress [19,20]. Greenleaf et al. [20] found that students having healthy cardiorespiratory fitness is associated with improved self-esteem and lessened depressive symptoms. In addition, studies have reported that children aged 7–12 years, who had a healthy cardiorespiratory fitness performed better on cognitive tests compared with their unfit peers [19,21]. A recent study provided a preliminary evidence that physical fitness has a direct effect on executive functions among youth [22]. However, there is still a paucity of studies on the association of cardiorespiratory fitness with PWB in childhood.

Promotion of PWB is an emerging social, educational, and health objective, especially for school-aged children. To the best of our knowledge, little is known about whether and to what degree cognitive function and cardiorespiratory fitness contribute to PWB in school-aged children. Thus, the purpose of this study was to examine associations of cardiorespiratory fitness and cognitive function (i.e., concentration performance, attention span, and attention accuracy) with PWB in fourth-grade students. The significance of the study was that results of the investigation would provide insightful information for informing the design of effective intervention strategies for promoting mental health and well-being in youth.

## 2. Methods

### 2.1. Participants

The study participants were 752 fourth-grade students (Mean _age_ = 9.61 years, SD = 0.608), recruited from six elementary schools. To actively recruit the participants, we sent an invitation letter along with consent form to each school principal. They reviewed the study protocols and granted approval for this study. We invited physical education (PE) teachers from the six elementary schools to attend the training in recruitment procedures and protocols for the study-related tests and questionnaires. Students were eligible participants if they met all inclusion criteria, including: (a) enrolled, fourth-grade students; (b) typically developed students without physical, cognitive, or mental impairments; and (c) consent/assent to complete all study-related questionnaires and tests. Students were not eligible to participate if they met one of the exclusion criteria, including (a) declined to participate, (b) students’ parent/legal guardian refused to allow their child(ren) to participate, or (c) could not complete study-related tests and questionnaire due to severe physical, cognitive, or mental impairment or disabilities, accidents, injuries, or illness. Researchers explained the study purpose, assurance of confidentiality, and voluntary participation to the students in fourth-grade classes during a regular PE lesson. The University Institutional Review Board (HUM00149529) approved the study protocols. Students who returned their parental signed consent form participated in the data collection as follows.

### 2.2. Outcome Measures

*Cardiorespiratory fitness.* The valid and reliable Progressive Aerobic Cardiovascular Endurance Run (PACER)^®^ test [23] was widely used to assess students’ cardiorespiratory fitness during a PE lesson in the school gymnasium due to its feasibility. The PACER is a multistage shuttle run test where students run back and forth across a 15-m distance within their own lane marked by cones before and/or on the sound of beep at a specified pace that gets faster each minute as many laps as they can. Students continue this running pattern until they fail to reach the line before the sound of beep for the second time.

Prior to the test, the trained PE teacher set up the testing area strictly following the testing protocols. The PE teacher explained and demonstrated the PACER testing directions to the students first. Then, the PE teacher organized the students into two groups to take the PACER test following the test directions. One group took the test at a time. The group of students’ performance in the PACER test was video recorded for trained investigators to record each student’s testing score correctly later. A student’s score on the test is the number of laps completed successfully. The age- and gender-specified cut-off criteria [23] was use to categorize participants into two groups: health fitness zone (HFZ; PACER lap ≥ 21) and need improvement (NI; PACER lap < 21) for both boys and girls.

**Figure 1:**
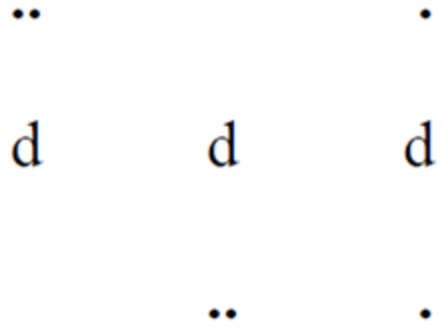
Examples of d2 mean [24].

*Cognitive function.* The d2 Test of Attention, a standardized, paper-and-pencil letter-cancellation test, was used to measure students’ cognitive function including concentration performance, attention span, and attention accuracy [24]. It consists of 14 lines of 47 randomly mixed letters “d” or “p” with 1–4 dashes arranged individually or in pairs above or below the character. The d2 Test allows 20 s per line to identify the letter “d” with two dashes, either above, below or one dash on top and one on the bottom (see Figure 1). Distractors are, more or less, dashes above or below the letter “d”, and two dashes or one dash above or below the letter “p” [24].

Three parameters of the d2 Test used in this study were: (a) concentration performance measures the capacity for focusing on stimuli without distraction. It is determined by subtracting the number of commission errors from the number of correct responses; (b) attention span measures the ability to sustain attention to stimuli. It is determined by using the line with the highest number of symbols processed minus the line having the lowest number of symbols processed; and (c) attention accuracy measures the ability to correctly respond to the stimuli. It is determined by using the combined number of omission and commission errors divided by the total number of the symbols processed. The d2 Test has high test-retest reliability coefficients for all parameters, ranging from 0.95 to 0.98. Test values for criterion, construct, and predictive validity have been stable over the course of 23 months after the initial testing in children [25].

Prior to administering the d2 Test, the trained research staff explained the testing directions to the students. Then, he/she asked the students to practice the two lines of the test on the front page of the standardized testing sheet following the testing directions. Next, the teacher administered the test to the entire class, using a stopwatch to give the verbal signal of “start”. When the teacher signaled “next” on every 20 s, the students changed to the next line to do so until finishing the last line. Students took ~5 min to complete the test.

*Psychological well-being.* The 14-item Warwick–Edinburgh Mental Well-Being Scale (WEMWBS) was used to measure students’ PWB, including positive affect (feelings of optimism, cheerfulness, relaxation) and positive functioning (energy, clear thinking, self-acceptance, personal development, competence and autonomy) [26]. The students were asked to circle the number that best reflected their experience of each statement in the past two weeks using a 5-point rating scale (0 = none of the time, 2 = rarely, 3 = some of the time, 4 = often, and 5 = all of the time). All 14 items were scored positively. The composite score of the 14 items was used to report levels of PWB. In previous studies, the scale had a Cronbach’s alpha value of 0.89 and had a test–retest reliability of 0.83 for a student sample aged 13–16 years [27].

Prior to administering the WEMWBS to the students, the trained research staff explained how to complete it to ensure all students understood how to do so. Then, the students began to complete the WEMWBS following the directions. In this study, the total scale had a Cronbach alpha coefficient of 0.90, indicating a high degree of internal consistency.

### 2.3. Data Analysis

Prior to conducting statistical analyses, a full data set, consisting of the PACER test, d2 Test, and the WEMWBS, was screened for missing values using a list-wise deletion, resulting in 35 cases deleted. Then, each case’s outliers were identified by using the SPSS_Explore method, resulting in 27 cases deleted. A final data set consisting of 689 cases was used for data analysis in this study. Descriptive statistics of each study variable were calculated for the total sample and by gender using IBM SPSS 27. Skewness and kurtosis of each variable were checked for normality. Each variable met the normality criteria for skewness between −2 to 2 and kurtosis between −7 to 7 indicating normality. [28]. Bivariate correlation coefficients between cardiorespiratory fitness and cognitive function (concentration performance, attention span, and attention accuracy) with PWB were performed to examine if there was a significant correlation between them. If so, then multiple linear regression models were performed to determine the extent to which cardiorespiratory fitness and cognitive functions (concentration performance, attention span, and attention accuracy) were associated with PWB, controlling for age, gender, and school. Multicollinearity for each independent variable was tested using tolerance (T) and variance inflation factor (VIF). The results of T for all independent variables were ranged from 0.734 to 0.998 (>0.01) and the values of VIF ranged from 1.002 to 1.363 (<5), an indication of no multicollinearity. Subsequently, standardized multiple regression coefficients were analyzed to assess a relative importance of each independent variable predicting PWB. Further, the mean score of PWB was used to classify the participants into high PWB group (above the average) and low PWB group (below the average group). Independent sample *t*-tests (Welch’s test was chosen due to unequal variance) were performed to examine if there was any significant difference in cardiorespiratory fitness, concentration performance, attention span, and attention accuracy between high PWB group and low PWB group. A significance level of *p* < 0.05 was set for all statistical methods.

## 3. Results

### 3.1. Preliminary Analysis

Table 1 presents the descriptive statistics of each variable. For the PACER test, less than one-fourth of the students’ cardiorespiratory fitness (21.2%) were in the HFZ. The mean scores of the PACER test in the total sample and boys and girls were lower than the low end of HFZ (21 laps). Regarding concentration performance, higher scores represent better performance. In contrast, for attention span and attention accuracy, lower scores indicate better performance. Based on the norms of attention accuracy for boys and girls aged 9–10 years [24], boys’ mean score (5.68) and girls’ mean score (5.41) were close to 50 percentile (5.6 = 50 percentile). With respects to PWB, according to the population norms of the PWB total score, a range of score: 40–59 is average [26]. The total sample’s mean score was in the range of average. Both the boy and the girl groups had similar mean scores.

### 3.2. Association of Cardiorespiratory Fitness and Cognitive Function with PWB

Table 2 presents bivariate correlation between the study variables. As seen in Table 2, PWB was positively associated with cardiorespiratory fitness (*p* < 0.01) and concentration performance (*p* < 0.05) and negatively related to attention span (*p* < 0.05), and attention accuracy (*p* < 0.01).

Due to the significant correlates of PWB, cardiorespiratory fitness, concentration performance, attention span, and attention accuracy were entered into the multiple linear regression model as the independent variables predicting PWB, the dependent variable (see Table 3). The results of the multiple linear regression model showed that the four independent variables were collectively significant predictors of PWB, accounting for 12% of the total variance in the PWB when controlling for age, gender, and school. Subsequently, the standardized regression coefficients (*β*) indicated that cardiorespiratory fitness was the most significantly individual predictor of the PWB, followed by attention accuracy.

### 3.3. Differences in Cardiorespiratory Fitness and Cognitive Function between High- and Low-PWB Groups

Table 4 presents the mean scores of cardiorespiratory fitness, concentration performance, attention span, and attention accuracy between high- and low-PWB groups. The results of the Welch’s test indicated that the high-PWB group scored significantly higher in cardiorespiratory fitness compared with the low-PWB group (*t* = 4.093, *df* = 685.741, *p* = 0.000, Cohen’s *d* = 0.310, 95% CI lower = −3683, upper = −1.295). Regarding the three parameters of cognitive function, the high-PWB group significantly outperformed than the low-PWB group in concentration performance (*t* = 3.340, *df* = 648.521, *p* = 0.001, Cohen’s *d* = 0.256, 95% CI lower = −16.679, upper = −4.329) and attention accuracy (*t* = −2.958, *df* = 618.007, *p* = 0.003, Cohen’s *d* = 0.130, 95% CI lower = 0.439, upper = 2.174). In addition, the high-PWB group showed better performance in attention span than the low-PWB group at a marginal significant level (*t* = −1.690, *df* = 641.241, *p* = 0.092, Cohen’s *d* = 0.229, 95% CI lower = −0.206, upper = 2.754).

## 4. Discussion

This is the first study that focuses on examining association of cardiorespiratory fitness and cognitive function with psychological well-being (PWB) in school-aged children. The findings indicate that the children’s cardiorespiratory fitness, concentration performance, attention span, and attention accuracy are collectively significant predictors of PWB. Among these independent variables, cardiorespiratory fitness is the most significantly individual contributor predicting PWB. Furthermore, the fourth-grade students with a higher level of PWB had better cardiorespiratory fitness than their counterparts who have a lower level of PWB.

Our unique results indicate that better cardiorespiratory fitness is a critical correlate and determinant of PWB in school-aged children. One possible reason for the essential role cardiorespiratory fitness played in PWB might be related to its mutual relationship with physical activity. Cardiorespiratory fitness is one of the enabling factors contributing to regular physical activity [22,29]. Especially, cardiorespiratory fitness is the most significant contributor for school-aged children to participate in a variety of physical activities and sports compared with the other enabling factors [22,30]. Children who are more physically fit have foundational physical conditioning required and demanded for successful participation in different intensity levels of physical activity and sports [31]. Thus, they are more likely to enjoy physical activity and sports and maintain their interests in them. Further, playing sports and engaging in physical activities, in turn, improve cardiorespiratory fitness level and maintain its healthy level [29]. Cardiorespiratory fitness and regular physical activity are mutually related and enhanced by each other. Given the well-established relationship between physical activity and PWB [14], our unique result, that is, cardiorespiratory fitness is a key correlate of PWB in school-aged children, is not surprising.

Another possible reason is that cardiorespiratory fitness is a key indicator of physical and mental health. In a conceptual model, Blair and colleagues [19] stress that physical fitness, particularly cardiorespiratory fitness, is a direct determinant of health outcomes. Children with healthy cardiorespiratory fitness are more likely to have and maintain healthy body weight and to have positive self-concept about their body image [20]. Children who have healthy cardiorespiratory fitness have a strong heart and a great capacity of maximal oxygen consumption, a good indicator of cardiovascular health [7,18]. Moreover, healthy cardiorespiratory fitness has produced beneficial effects on increasing cerebral blood volume flow, increasing growth of capillaries, and transporting nutrients to the brain [14,21]. The evidenced benefits of healthy cardiorespiratory fitness in physical health are instrumental for persons to possess sufficient energy, vitality, and the stamina necessary for effective functioning in their daily life [7,18]. Therefore, healthy cardiorespiratory fitness provides the physical foundation needed for positive mental health and psychological well-being in school-aged children.

However, it is important to note that in this study less than one-fourth of the children were in the HFZ of cardiorespiratory fitness. More concerning is that both boys’ and girls’ mean scores of PACER test were much lower than the cut-off point of 21 laps for meeting the HFZ, especially with even lower mean score of girls’ PACER test. Given the critical role cardiorespiratory fitness plays in contributing to PWB and the prevalence of an unhealthy level of cardiorespiratory fitness, this study suggests that school physical activity/education programs should focus on improving or maintaining cardiorespiratory fitness in the pre-adolescence years. Future intervention studies should design developmentally appropriate, fun, and age-appropriate aerobic physical activities in and outside of school settings for school-aged children. In particularly, this study suggests that improving girls’ cardiorespiratory fitness level should be one of the primary intervention strategies. This is because our study found that meeting the HFZ of cardiorespiratory fitness is a more challenging and urgent task for girls, compared with boys for this age group.

Another unique finding shows that students’ concentration performance, attention span, and attention accuracy are significantly collective predictors of PWB. Among the correlates of PWB, attention accuracy is a significantly individual predictor of PWB. Moreover, the fourth-grade students who have a better level of PWB exhibit a better level of concentration performance and attention accuracy. Conversely, the children with a lower level of PWB have a lower level of concentration performance and attention accuracy. Concentration performance indicates an individual’s ability to choose what they focus on while ignoring irrelevant stimuli. Attention accuracy reflects an individual’s selective attention and inhibition control [15,25]. Concentration performance and attention accuracy directly impact students’ ability to concentrate on what they learn, to accurately learn new information, to correctly complete tasks, and to find right solutions to problems [15,16]. A high level of concentration performance and attention accuracy are key determinants of academic performance and achievement. For school-aged children, their academic performance and achievement positively influence their PWB such as positive affect, self-acceptance, and competency [14].

The current profile of the students’ attention and concentration performance provides possible explanation for the underlying mechanism. The present students display a promising level of attention and concentration. Their performance is centering on 50 percentile category compared to the population norms [24]. In addition, both boys and girls of the study demonstrate an overall high level of concentration performance and attention accuracy compared to a study by Buchele et al. [15]. With respect to concentration performance, the present students’ mean score was 135.27. In contrast, the fifth-grade students’ mean score was 100.11 in Buchele et al.’s study. Regarding attention accuracy, the present students’ mean score was 5.55, while in Buchele et al.’s study [15] the fifth-grade students exhibit a mean score of 7.77. The lower the mean score of attention accuracy, the better performance the attention accuracy indicates [24]. This study suggests that future intervention strategies should focus on developing and strengthening students’ ability to accurately focus on what they are learning and doing in order to promote PWB in childhood. The strength of our study is providing empirical evidence that students’ healthy cardiorespiratory fitness and their ability to focus and concentrate accurately are key correlates of PWB.

The study has several limitations. First, the study only focused on examining the association of cardiorespiratory fitness and attention and concentration performance with PWB. Future study should explore how well others factors, such as positive personal attributes, demographic characteristics, and physical activity participation, are associated with PWB. Second, this study did not investigate a mediating role of attention and concentration performance in the relationship between cardiorespiratory fitness and PWB or vice versa. Examining potential mediators in the relationship between cardiorespiratory fitness and PWB should be warranted in future studies in order to better understand the underlying mechanism. Lastly, due to the nature of the cross-sectional design, the causal link of cardiorespiratory fitness and cognitive function to PWB cannot be concluded. However, the present results may inform future health interventions for targeting the promotion of PWB—they should focus on improving cardiorespiratory fitness and attention and concentration among school-aged children.

In conclusion, our study found that cardiorespiratory fitness and cognitive function (i.e., concentration performance, attention span, and attention accuracy) play essential roles in contributing to PWB in school-aged children. Further, cardiorespiratory fitness and attention accuracy were significantly individual predictors of PWB. Students with a higher level of PWB exhibit a better level of cardiorespiratory fitness and a higher level of concentration performance and attention accuracy. In contrast, students with a lower level of PWB have a lower level of cardiorespiratory fitness and a lower level of concentration performance and attention accuracy. The study suggests that future intervention strategies should focus on improving cardiorespiratory fitness and cognitive function along with other correlates to promote PWB, in turn, improving overall physical and mental health in school-aged children.

## Figures and Tables

**Table 1:** Descriptive statistics of the variables for the total sample and by gender.

Variables	Total (*n* = 689)	Boys (*n* = 370)	Girls (*n* = 319)
	Mean (SD)	Mean (SD)	Mean (SD)
age	9.63 (0.621)	9.67 (0.659)	9.58 (0.582)
PACER in HFZ *n* (%)	146 (21.2%)	97 (26.2%)	49 (15.4%)
PACER	16.52 (18.283)	17.60 (9.407)	15.49 (6.467)
concentration	135.27 (41.356)	134.75 (41.451)	135.87 (41.303)
attention span	18.28 (9.815)	19.04 (10.135)	17.39 (9.369)
attention accuracy	5.55 (5.729)	5.68 (5.846)	5.41 (5.595)
PWB (total score)	52.37(10.984)	52.11 (10.948)	52.51 (10.848)

Notes. SD = standard deviation PACER = progressive aerobic cardiovascular endurance run; HFZ = healthy fitness zone; PWB = psychological well-being.

**Table 2:** Bivariate correlation among the study variables.

Measure	1	2	3	4	5
1. PWB	-				
2. concentration	0.092 *	-			
3. attention span	−0.069 *	−0.105 **	-		
4. attention accuracy	−0.103 **	−0.406 **	−0.325 **	-	
5. cardiorespiratory fitness	0.161 **	−0.075 *	−0.007	0.022	-

Notes. PWB = psychological well-being; * = *p* < 0.05; ** = *p* < 0.01.

**Table 3:** Results of multiple linear regression model for predicting psychological well-being.

Variable	R	R^2^	F	*p*	df	Beta	t	*p*
model	0.347	0.120	13.299	0.000	(6.682)			
cardiorespiratory fitness						0.174	4.733	0.000
concentration						0.002	0.054	0.957
attention span						0.005	0.123	0.902
attention accuracy						−0.090	−2.156	0.031

**Table 4:** Mean scores of cardiorespiratory fitness and cognitive function between high and low PWB groups.

Outcome Variables	Low-PWB Group (*n* = 298)	High-PWB Group (*n* = 391)
	Mean (SD)	Mean (SD)
cardiorespiratory fitness ***	15.21 (7.070)	17.70 (8.889)
concentration ***	129.31 (40.368)	139.82 (41.574)
attention span ***	19.00 (9.777)	17.73 (9.827)
attention accuracy *	6.30 (5.896)	4.99 (5.539)

Notes. PWB = psychological well-being; SD = standard deviation; *** = *p* < 0.01; * = *p* < 0.10.

## Data Availability

The data that support the findings of this study are available on request from the corresponding author [WC]. The data are not publicly available due to them containing information that could compromise the privacy of research participants.

## References

[1] Ryff C.D. (2014). Psychological well-being revisited: Advances in the science and practice of eudaimonia. Psychother. Psychosom..

[2] Steptoe A., Deaton A., Stone A.A. (2015). Psychological wellbeing, health and ageing. Lancet.

[3] World Health Organization (WHO) (2014). Mental Health: A State of Well-Being.

[4] Dodge R., Daly A.P., Huyton J., Sanders L.D. (2012). The challenge of defining wellbeing. Int. Lournal Wellbeing.

[5] Liddle I., Carter G.F. (2015). Emotional and psychological well-being in children: The development and validation of the Stirling Children’s Well-being Scale. Educ. Psychol. Pract..

[6] Boehm J.K., Kubzansky L.D. (2012). The heart’s content: The association between positive psychological well-being and cardiovascular health. Psycholocial Bull..

[7] Ortega F.B., Lee D.-C., Sui X., Kubzansky L.D., Ruiz J.R., Baruth M., Castillo M.J., Blair S.N. (2010). Psychological well-being, cardiorespiratory fitness, and long-term survival. Am. J. Prev. Med..

[8] Zaslavsky O., Rillamas-Sun E., Woods N.F., Cochrane B.B., Stefanick M.L., Tindle H., Tinker L.F., LaCroix A.Z. (2014). Association of the selected dimensions of eudaimonic well-being with healthy survival to 85 years of age in older women. Int. Psychogeriatr..

[9] Sin N.L. (2016). The protective role of positive well-being in cardiovascular disease: Review of current evidence, mechanisms, and clinical implications. Curr. Cardiol. Rep..

[10] Reschly A.L., Huebner E.S., Appleton J.J., Antaramian S. (2008). Engagement as flourishing: The contribution of positive emotions and coping to adolescents’ engagement at school and with learning. Psychol. Schools.

[11] Suldo S., Thalji A., Ferron J. (2011). Longitudinal academic outcomes predicted by early adolescents’ subjective well-being, psychopathology, and mental health status yielded from a dual factor model. J. Posit. Psychol..

[12] Dockray S., Steptoe A. (2010). Positive affect and psychobiological processes. Neurosci. Biobehav. Rev..

[13] Trudel-Fitzgerald C., Millstein R.A., von Hippel C., Howe C.J., Tomasso L.P., Wagner G.R., VanderWeele T.J. (2019). Psychological well-being as part of the public health debate? Insight into dimensions, interventions, and policy. BMC Public Health.

[14] Lubans D., Richards J., Hillman C., Faulkner G., Beauchamp M., Nilsson M., Kelly P., Smith J., Raine L., Biddle S. (2016). Physical activity for cognitive and mental health in youth: A systematic review of mechanisms. Pediatrics.

[15] Buchele Harris H., Cortina K.S., Templin T., Colabianchi N., Chen W. (2018). Impact of coordinated-bilateral physical activities on attention and concentration in school-aged children. Biomed Res. Int..

[16] Janssen M., Toussaint H.M., van Mechelen W., Verhagen E.A. (2014). Effects of acute bouts of physical activity on children’s attention: A systematic review of the literature. SpringerPlus.

[17] Llewellyn D.J., Lang I.A., Langa K.M., Huppert F.A. (2008). Cognitive function and psychological well-being: Findings from a population-based cohort. Age Ageing.

[18] Raghuveer G., Hartz J., Lubans D.R., Takken T., Wiltz J.L., Mietus-Snyder M., Perak A.M., Baker-Smith C., Pietris N., Edwards N.M. (2020). Cardiorespiratory fitness in youth: An important marker of health: A scientific statement from the American heart association. Circulation.

[19] Blair S.N., Cheng Y., Holder J.S. (2001). Is physical activity or physical fitness more important in defining health benefits?. Med. Sci. Sports Exerc..

[20] Greenleaf C.A., Petrie T.A., Martin S.B. (2010). Psychosocial variables associated with body composition and cardiorespiratory fitness in middle school students. Res. Q. Exerc. Sport..

[21] Buck S.M., Hillman C.H., Castelli D.M. (2008). The relation of aerobic fitness to stroop task performance in preadolescent children. Med. Sci. Sports Exerc..

[22] Gu X., Zhang T., Lun Chu T., Zhang X., Thomas Thomas K. (2019). Do physically literate adolescents have better academic performance?. Percept. Mot. Skills.

[23] The Cooper Institute (2017). FitnessGram Administration Manual: The Journey to MyHealthyZone.

[24] Brickenkamp R. (2002). The d2 Test of Attention 10th Expanded and Revised Edition.

[25] Bates M.E., Lemay E.P. (2004). The d2 Test of attention: Construct validity and extensions in scoring techniques. J. Int. Neuropsychol. Soc..

[26] Tennant R., Hiller L., Fishwick R., Platt S., Joseph S., Weich S., Parkinson J., Secker J., Stewart-Brown S. (2007). The Warwick-Edinburgh mental well-being scale (WEMWBS): Development and UK validation. Health Qual. Life Outcomes.

[27] Clarke A., Friede T., Putz R., Ashdown J., Martin S., Blake A., Adi Y., Parkinson J., Flynn P., Platt S. (2011). Warwick-Edinburgh Mental Well-being Scale (WEMWBS): Validated for teenage school students in England and Scotland. A mixed methods assessment. BMC Public Health.

[28] Hair J.F., Black W.C., Babin B.J., Anderson R.E. (2010). Multivariate Data Analysis.

[29] Stodden D.F., Goodway J.D., Langendorfer S.J., Roberton M.A., Rudisill M.E., Garcia C., Garcia L.E. (2008). A developmental perspective on the role of motor skill competence in physical activity: An emergent relationship. Quest.

[30] Castelli D.M., Hillman C.H., Buck S.M., Erwin H.E. (2007). Physical fitness and academic achievement in third- and fifth-grade students. J. Sport Exerc. Psychol..

[31] Chen W.Y., Hammond-Bennett A., Hypnar A., Mason S. (2018). Health-related physical fitness and physical activity in elementary school students. BMC Public Health.

